# Supplementary material for: Causal interplay between lactose intolerance and gut microbiota: a combined bidirectional Mendelian randomization and in vivo validation study
Source: Front Nutr. 2026 Jun 1;13:1803337. doi: 10.3389/fnut.2026.1803337 (PMC13265576; doi:10.3389/fnut.2026.1803337)
Supplement: Supplementary file 1 [file Data_Sheet_1.zip › supplementary materials/Forward/leave one out cross validation/ebi-a-GCST90027544.finngen_R12_E4_LACTONAS.pdf]

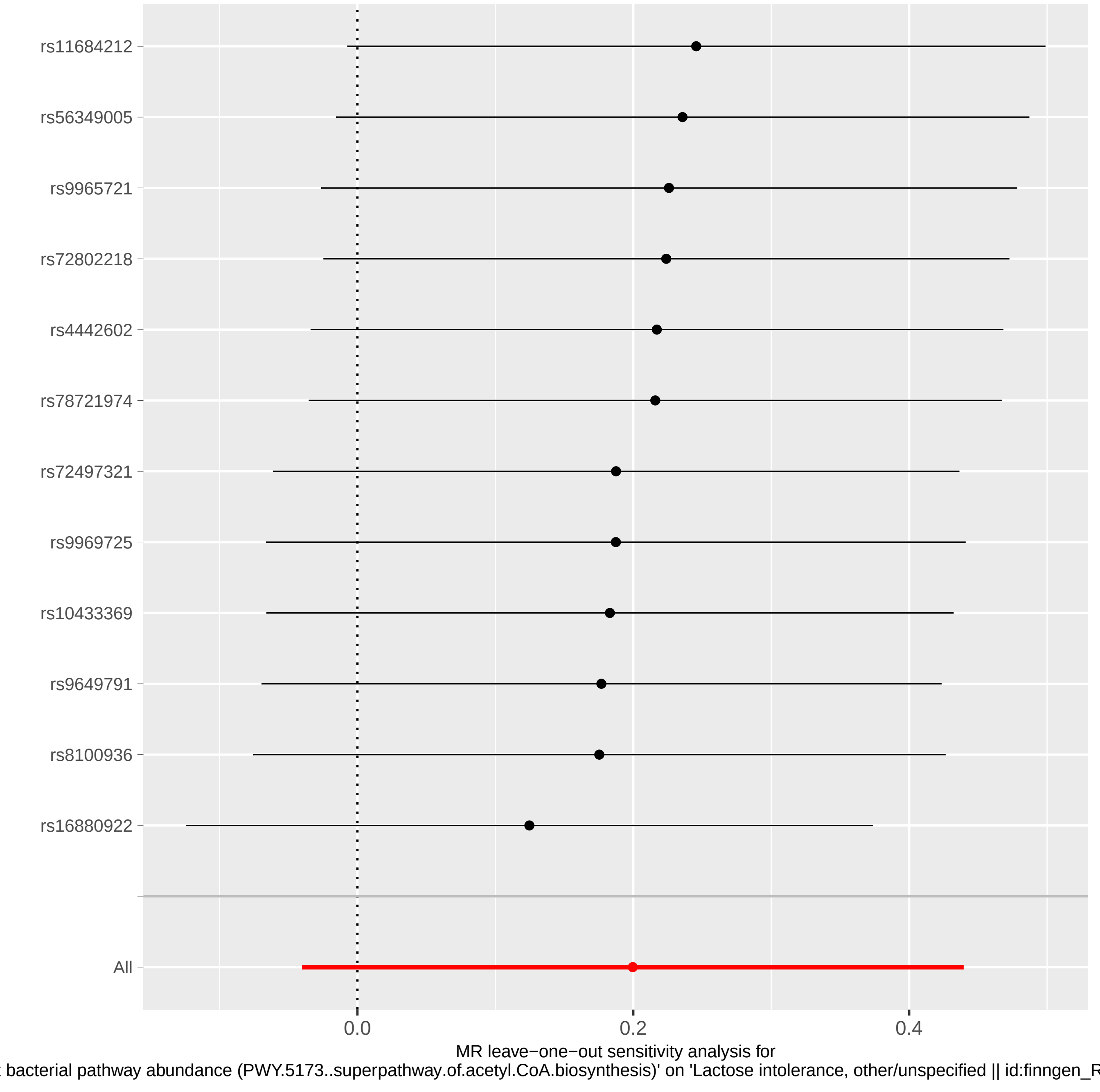
rs11684212
rs56349005
rs9965721
rs72802218
rs4442602
rs78721974
rs72497321
rs9969725
rs10433369
rs9649791
rs8100936
rs16880922
All
0.0
0.2
0.4
MR leave−one−out sensitivity analysis for
bacterial pathway abundance (PWY.5173..superpathway.of.acetyl.CoA.biosynthesis)' on 'Lactose intolerance, other/unspecified || id:finngen_R
